# Statistical relationship between metabolic decomposition and chemical uptake predicts bioconcentration factor data for diverse chemical exposures

**DOI:** 10.1186/s12918-018-0601-y

**Published:** 2018-08-07

**Authors:** Michael A. Rowland, Hannah Wear, Karen H. Watanabe, Kurt A. Gust, Michael L. Mayo

**Affiliations:** 1 Environmental Laboratory, US Army Engineer Research and Development Center, Vicksburg, MS USA; 2 Oak Ridge Institute for Science and Education, Oak Ridge, TN USA; 3 Portland State University, Portland, OR USA; 4 School of Mathematical and Natural Sciences, Arizona State University, Glendale, AZ USA

**Keywords:** IVIVE, Physiologically based toxicokinetics, Reverse toxicokinetics, Bioconcentration factors

## Abstract

**Background:**

A challenge of in vitro to in vivo extrapolation (IVIVE) is to predict the physical state of organisms exposed to chemicals in the environment from in vitro exposure assay data. Although toxicokinetic modeling approaches promise to bridge in vitro screening data with in vivo effects, they are often encumbered by a need for redesign or re-parameterization when applied to different tissues or chemicals.

**Results:**

We demonstrate a parameterization of reverse toxicokinetic (rTK) models developed for the adult zebrafish (*Danio rerio*) based upon particle swarm optimizations (PSO) of the chemical uptake and degradation rates that predict bioconcentration factors (BCF) for a broad range of chemicals. PSO reveals a relationship between chemical uptake and decomposition parameter values that predicts chemical-specific BCF values with moderate statistical agreement to a limited yet diverse chemical dataset, and all without a need to retrain the model to new data.

**Conclusions:**

The presented model requires only the octanol-water partitioning ratio to predict BCFs to a fidelity consistent with existing QSAR models. This success begs re-evaluation of the modeling assumptions; specifically, it suggests that chemical uptake into arterial blood may be limited by transport across gill membranes (diffusion) rather than by counter-current flow between gill lamellae (convection). Therefore, more detailed molecular modeling of aquatic respiration may further improve predictive accuracy of the rTK approach.

**Electronic supplementary material:**

The online version of this article (10.1186/s12918-018-0601-y) contains supplementary material, which is available to authorized users.

## Background

How can data from high-throughput in vitro assays be used to assess the ecological effects of chemicals before they are released into the environment? One promising approach, referred to as in vitro to in vivo extrapolation (IVIVE), aims to approximate the in vivo effects of exposure to xenobiotic substances entirely from in vitro data that are generated quickly and inexpensively. This grand challenge is being addressed by the Toxicology in the 21^st^ Century (Tox21) program [1], which promises to develop rapid toxicity assessment protocols to better evaluate how chemicals impact public health. Most in vitro data simply do not account for the absorption, distribution, metabolism, and excretion (ADME) of chemicals throughout the body, and, therefore, may not accurately reflect in vivo responses [2]. Physiologically based toxicokinetic (PBTK) in addition to reverse toxicokinetic (rTK, alternatively termed exposure reconstruction or reverse dosimetry) modeling have largely been leveraged to bridge this gap between in vitro data and in vivo responses. Unfortunately, a lack of species-specific standardization among PBTK/rTK models ensures that new iterations are developed on a case-by-case basis [3–15], contributing to a wealth of PBTK/rTK approaches which lack the generality necessary to equally handle the large amount of chemical-specific bioactivity data gathered from high-throughput assays, such as those available from ToxCast [16, 17].

Another issue limiting the generality and standardization of existing IVIVE approaches is that decisions affecting the scale of a compartment-based physiological model can strongly influence its predictions. For example, rTK modeling is used to predict an exposure concentration associated with a given tissue-specific, internal steady-state concentration measurement [18]. However, we have previously shown how the number and connectivity of compartments representing the different tissues and organs of the body affect the relative accuracy of PBTK/rTK modeling predictions for aquatic organisms. Specifically, we found that a more complex rTK approach, which models the body at a fine scale (higher physiology fidelity), consistently underestimates exposure concentration predictions when contrasted against predictions from more frugal single-compartment models (lower physiological fidelity) for the same organism and identical whole-body tissue concentrations [19]. Our previous analytical results stand in contrast to previous results showing a lower predicted body concentration by a multi-compartment PBTK model when compared to a one-compartment model [20]. We must note, however, that this multi-compartment PBTK model has all compartments in parallel without any interactions between any of them; our previous results would suggest that this would provide predictions similar to that of a one-compartment model [19].

In this work we investigate the ability of rTK models, built with varying physiological fidelity to the teleost zebrafish (*Danio rerio*), to predict bioconcentration factors (BCFs) associated with a large sampling of chemicals without chemical-specific model retraining. For these tasks we leverage two previously developed rTK models: a simple model representing zebrafish as a single compartment, and a more complex model that partitions the body into 7 interconnected compartments [19]. This single compartment model is only mildly successful in predicting BCFs for a broad range of chemicals and consistently underestimates BCF measurement data. Using the 7-compartment model we show that choosing median values for only two parameters—a first-order rate constant that we will refer to as “metabolic decomposition” or “degradation rate” represents xenobiotic decomposition by liver metabolism, biotransformation, and excretion; and another constant that quantifies water flow across the exchange surface of gill lamellae—produces accurate BCF predictions when given only the octanol-water partition ratio as the identifying chemical information. Finally, we explore the extent to which these models, optimized using adult zebrafish data, are predictive of BCFs across different life-stages or for other fish species. We generally find that our adult models generally should not be used to predict zebrafish embryo BCFs. However, the adult zebrafish models are predictive of BCFs within adults of a subset of other species when compared against data collected across a range of fish species.

## Results

### Optimizing reverse toxicokinetic models for BCF predictions

**Fig. 1:**
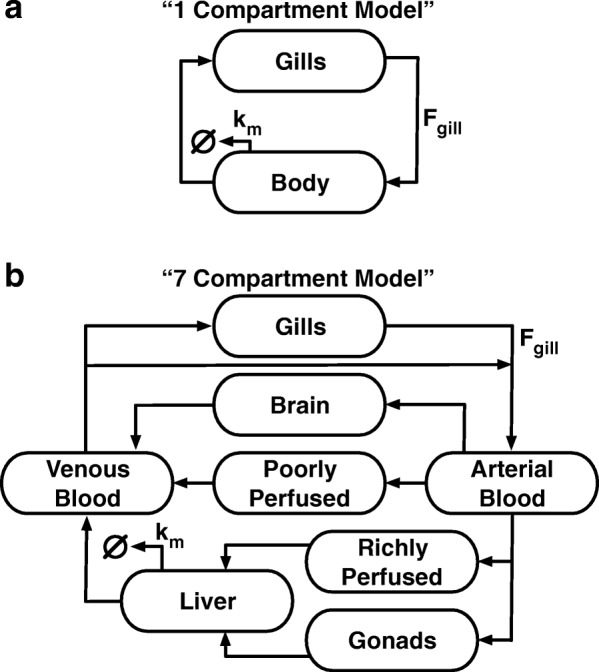
Diagrams of the models. **a** The “1-Compartment” (1C) model, abstracting the adult zebrafish to a single compartment with gills. Chemical enters the body through the gills. Some of the chemical is then metabolized, some is retained within the body, and the rest exits back through the gills. **b** The “7-Compartment” (7C) model breaks the zebrafish up into arterial blood, venous blood, brain, gonads, poorly perfused tissue, richly perfused tissue, and liver. Chemical enters the body through the gills, initially accumulating in the arterial blood. The arterial blood then distributes chemical to each of the body compartments, except for the venous blood. The richly perfused tissues and gonads empty directly into the liver; the other compartments empty into the venous blood. The liver also can metabolize the chemical. Chemical in the venous blood either recirculates into the arterial blood or leaves the body through the gills

Suppose that two rTK models, a 1-compartment (1C) model of lower physiological fidelity and a multi-compartment model of higher fidelity, which distinguishes between zebrafish organs/tissues, are given identical whole-body concentrations as input to predict the exposure concentrations that would lead to such a degree of bioaccumulation. Will one model produce more accurate predictions than the other because of their differing complexity? Can their predictive accuracy be improved by adjusting one or more parameter values? The 1C model used here to address these questions is similar to previously published human PBTK models wherein the body is treated as a well-mixed single compartment chemical-reactor (Fig. 1a) [19, 21, 22]. We offset the relative simplicity of the 1C model with a more complex model that splits zebrafish into 7 interconnected compartments (7C), including arterial and venous blood, brain, gonads, liver, poorly perfused tissue and richly perfused tissue, and is based on a previously published PBTK model of zebrafish (Fig. 1b) [11, 19]. These 1C and 7C TK models accept aqueous concentrations as input to predict aggregate tissue concentrations within the organism. They were each analytically inverted to produce a rTK model by assuming an exposure medium of homogeneously distributed, and constant concentration of dissolved chemical so that its distribution throughout the body can be considered at steady-state. The result is an analytical expression for the predicted exposure concentration, *C*_*exp*_, as a function of the body concentration, *C*_*body*_ (See Additional file 1, Section “Results” for details). This relationship takes the form:

1 $$ {C}_{\mathrm{exp}}=\frac{C_{body}}{BCF} $$

wherein *BCF* is a ratio of the tissue to aqueous concentrations at steady state, and can be predicted by the models based solely on chemical-independent body parameters (e.g. lipid contents, water contents, and blood flow rates) and a single chemical-dependent octanol-water ratio, log_10_*K*_ow_.

**Table 1:** List and description of physiological parameters in the 7C model that are allowed to vary in either the all parameter PSOs and the source/sink PSOs

Parameter	Description	All Parameter PSO	Source/Sink PSO
F_gill_	Water Flow Rate Through Gills	*	*
k_m_	Metabolic Decomposition Rate	*	*
f_car_	Total Blood Flow Rate	*	
f_brn_	Percentage of Blood Flow to Brain	*	
f_gon_	Percentage of Blood Flow to Gonads	*	
f_rpt_	Percentage of Blood Flow to Richly Perfused Tissues	*	
f_ppt_	Percentage of Blood Flow to Poorly Perfused Tissues	*	
f_liv_	Percentage of Blood Flow to Liver	*	
m_tot_	Total Mass	*	
m_bld_	Percentage of Mass in Blood	*	
m_brn_	Percentage of Mass in Brain	*	
m_gon_	Percentage of Mass in Gonads	*	
m_rpt_	Percentage of Mass in Richly Perfused Tissues	*	
m_ppt_	Percentage of Mass in Poorly Perfused Tissues	*	
m_liv_	Percentage of Mass in Liver	*	
L_brn_	Lipid Content of Brain	*	
L_gon_	Lipid Content of Gonads	*	
L_rpt_	Lipid Content of Richly Perfused Tissues	*	
L_ppt_	Lipid Content of Poorly Perfused Tissues	*	
L_liv_	Lipid Content of Liver	*	
W_brn_	Water Content of Brain	*	
W_gon_	Water Content of Gonads	*	
W_rpt_	Water Content of Richly Perfused Tissues	*	
W_ppt_	Water Content of Poorly Perfused Tissues	*	
W_liv_	Water Content of Liver	*	

Note that model parameters that are dependent on the log_10_*K*_*ow*_ ratio of the chemical (e.g. the tissue/blood partition ratios for each compartment) are not directly optimized but are altered indirectly by the PSOs through these parameters. See Additional file 1, section “Discussion” for further details on these parameters and the chemical-dependent parameters

*Indicates the inclusion of the indicated parameter in the optimization strategy

To evaluate the predictive capability of these models, we collected a dataset of 76 unique BCFs and octanol-water ratios. These values represent BCFs determined by exposing zebrafish to different chemicals in a flow-through setup [23–30]. The chemicals of this dataset include different polychlorinated biphenyls, anilines, phenols, and benzenamines. These data represent a broad range of log_10_*K*_*ow*_ values, from 0.8 (hydrophilic) to 8.48 (hydrophobic), in addition to log_10_ BCF values ranging from − 0.10 to 5.97 (Additional file 1: Figure S1 and Additional file 2: Table S1). We randomly split these data into two distinct sets of equal size, one that was used for model training and the other for validation. As explained in Materials and Methods, particle swarm optimizations (PSO) were used to identify parameter sets that improved model performance relative to training data.

**Fig. 2:**
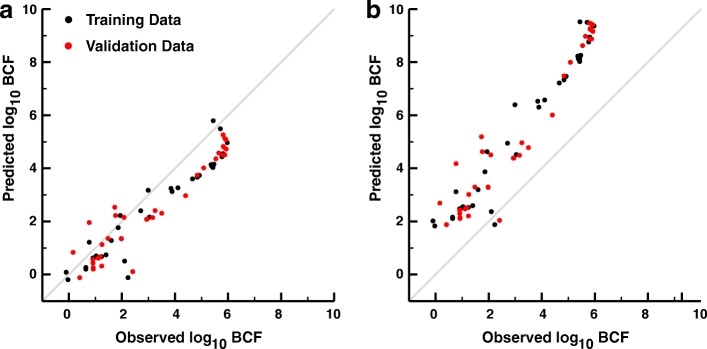
The rTK models with literature-derived parameter values. **a** The predicted log_10_ BCF values estimated by the 1C model from the log_10_*K*_*ow*_ values for each of the chemicals in the training (black dots) and validation (red dots) sets vs. the observed log_10_ BCF values using literature-derived parameter values. The grey line represents the line of equality (y=x). RMSE = 0.894 and 0.924 for the training and validation sets. **b** The predicted log_10_ BCF values estimated by the 7C model from the log_10_*K*_*ow*_ values for each of the chemicals in the training and validation sets vs. the observed log_10_ BCF values using literature-derived parameter values. RMSE = 2.331 and 2.360 for the training and validation sets

The 1C model performed modestly using literature-derived parameter values (RMSE = 0.894 and 0.924 for the training and validation datasets, respectively, Fig. 2a), and is given by the equation:

2 $$ BCF=\frac{P_{bw}}{1+\frac{V_t{k}_m}{F_{gill}{P}_t}{P}_{bw}} $$

Here, *F*_*gill*_ is the countercurrent water-flow rate between gill lamellae (μL s^− 1^), *k*_*m*_ is the rate of chemical degradation (s^− 1^), *V*_*t*_ is the total volume of the body, *P*_*bw*_ is the blood-water partition ratio, and *P*_*t*_ is the tissue-blood partition ratio. This model significantly underestimates most chemical BCFs for both training and validation datasets (66/76 underestimated, *p* = 2.96 × 10^− 11^, binomial test). PSOs of all physiological parameters provided no improvement in model accuracy (See Additional file 1, section “Results”).

PSOs tended to drive the degradation rate toward 0 over many successive updating steps, which is curious because liver metabolism is one of only two chemical sinks built into the model. Its consequences can be investigated analytically by calculating the limit using Eq. (2):

3 $$ \underset{k_m\to 0}{\lim } BCF={P}_{bw} $$

For a BCF > 1 with *P*_*bw*_ > 1, *k*_*m*_ ought to be bounded from above by ${k}_m<\frac{F_{gill}{P}_t}{V_t}\left(1-\frac{1}{P_{bw}}\right)$ in any optimization strategy according to Eq. (2). However, using Eq. (3) in place of Eq. (2) results in only in a small (nearly indiscernible) increase in predictive agreement (Additional file 1: Figure S2, RMSE = 0.909 across full dataset). Our rTK models describe blood-water partitioning in terms of only the octanol-water partition ratio:

log_10_*P*_*bw*_ = 0.78 log_10_*K*_*ow*_ – 0.82 [11].

Thus, *P*_*bw*_ < 1 is possible for hydrophilic chemicals, i.e., log_10_*K*_*ow*_ < 1.05. Under such extreme circumstances it is impossible for a *k*_*m*_ value to improve BCF agreement with experimental data, which suggests that the 1C model may underestimate the BCF for chemical with log_10_*K*_*ow*_ < 1.05.

In contrast to the 1C model, the 7C model significantly overestimated chemical-dependent BCF predictions using only literature values as input for body parameters (Fig. 2b, RMSE = 2.331 and 2.360 for the training and validation data sets, 74/76 BCFs overestimated, *p* < 2.2 × 10^− 16^, binomial test). It was expected for the 7C model to overestimate the BCFs relative to the 1C model; we had previously demonstrated that, given the same whole-body chemical concentrations, the 1C consistently overestimated the exposure concentrations compared to the 7C model [19]. Optimizing all physiological parameters in the 7C model (see Table 1) results in an increase in model accuracy (RMSE = 0.728 and 0.850 for training and validation sets), but at the cost of the biological relevance. The optimized parameters depict an organism with disproportionately sized tissues and a lower lipid content – a system poorly suited for the accumulation of lipophilic chemicals. (See Additional file 1 section “Results” for details). Therefore, in the following efforts, we explored the optimizations for more conceptually and empirically challenging parameters, such as xenobiotic decomposition and flux across the gill while keeping well-established parameters restricted to biologically relevant values.

**Fig. 3:**
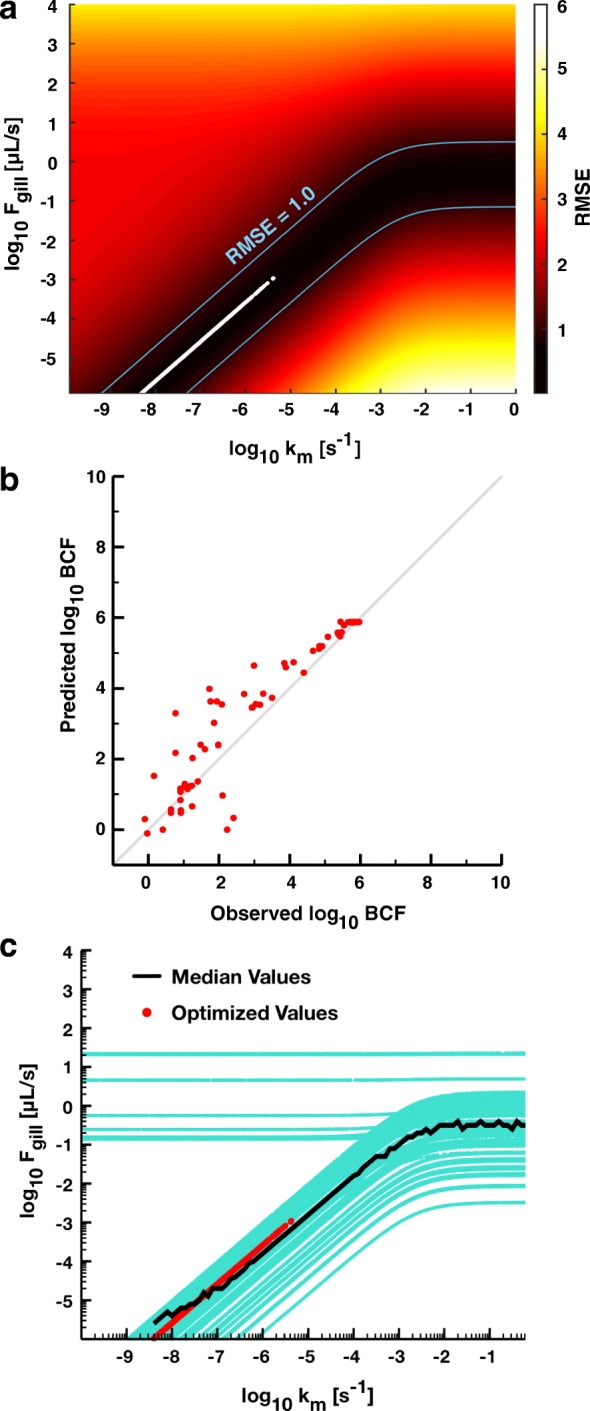
Optimization of the 7C model’s source and sink parameters. **a** The heatmap represents the RMSE value of the 7C model using literature-derived values for the body parameters with the exception of *F*_*gill*_ and *k*_*m*_, which are set according to the values on their respective axes. RMSE values are calculated based on the predicted log_10_ BCF vs. observed log_10_ BCF for all 76 chemicals in the adult zebrafish dataset. The blue lines represent the values of *F*_*gill*_ and *k*_*m*_ that parameterize the 7C model to predict the BCFs with RMSE = 1.0. The white dots represent the resulting parameter values from 1000 PSOs optimizing only *F*_*gill*_ and *k*_*m*_. **b** The predicted log_10_ BCF values estimated by the 7C model from the log_10_*K*_*ow*_ values for each of the chemicals in the training and validation sets vs. the observed log_10_ BCF values after optimization of the *F*_*gill*_ and *k*_*m*_ values. **c** The blue dots represent the resulting parameter values from 1000 PSOs optimizing *F*_*gill*_ and *k*_*m*_ so that the 7C model accurately predicts each of the 76 chemicals independently (relative error of the predicted vs. observed log_10_ BCF < 10^− 3^). The black line represents the median value of *F*_*gill*_ for a range of *k*_*m*_ across all 76 chemical-specific optimizations. The red dots are the resulting parameter values from the 1000 PSOs optimizing *F*_*gill*_ and *k*_*m*_ from the 7C model for all chemicals

We used PSO to restrict See Table 1 for subset of source and sink parameters from the list of physiological parameters, because indiscriminate optimization of all model parameters led to physiologically implausible yet optimal parameter values. Xenobiotic substances enter fish primarily from respiration (source) regulated by the countercurrent water-flow rate across gill lamellae (*F*_*gill*_), and eliminated in liver tissues (sink) by the rate of first-order metabolic decomposition (*k*_*m*_) [11, 19]. Thus, body burden can be adjusted by finely tuning these two parameters in order to modulate accumulation from the aquatic environment. We modeled BCF data associated with all 76 chemical data points by varying *k*_*m*_ and *F*_*gill*_ while holding all others constant according to their literature-derived values. For each pair of parameter values, we calculated RMSE for points in the predicted vs. observed plane (Fig. 3a). We found a range of values for both *F*_*gill*_ and *k*_*m*_ that allow the 7C model to predict BCFs for all 76 chemicals with a high degree of accuracy (Fig. 3a, RMSE < 1 inside the blue contours). We used PSO to confirm these results by re-optimizing the model for only these two parameters 1000 times and starting from identical initial parameter values. All 1000 optimizations identified values for *F*_*gill*_ and *k*_*m*_ within the region of smallest RMSE values found previously, with an average of about 0.791 (Fig. 3a, white dots). We confirmed the models’ high fidelity by inspection after plotting the highest scoring optimization (RMSE = 0.791) in the predicted vs. observed plane of BCF values (Fig. 3b).

Based on these results, we conclude that just a single value for the rate of a chemical’s metabolic decomposition in liver tissues allows for a robust predictive environment for exposure reconstruction, which is surprising given that metabolic decomposition rates (e.g., biotransformation, conjugation, degradation) measured for even structurally similar chemicals can vary by orders of magnitude [24]. In addition, these results suggest that a common rate of metabolic decomposition may be empirically correlated with parameters of chemical uptake from aqueous media in a way that leaves overall model fidelity invariant. This result directly arises from the structure of the model as a change in the uptake rate would require a change in the decomposition rate to maintain the steady state body concentration. Testing this mechanistic hypothesis, however, would be an interesting future comparison between the mathematical models and biological activity.

### A reinterpretation of contaminant flux across the gill

Optimizing the 7C model to all chemicals with a universal rate of chemical decomposition trades the absolute predictive accuracy of any single chemical for improved predictions over the ensemble of chemical data, and begs the question of whether this approximation is biologically reasonable. To address this question we used PSOs to optimize the 7C model to each BCF datum individually by varying *F*_*gill*_ and *k*_*m*_ while keeping all other parameter values fixed, which results in a distribution of chemical-specific *F*_*gill*_ and *k*_*m*_ values. Particles in the PSO were scored based on the relative error, i.e., |log_10_ BCF_observed_ – log_10_ BCF_predicted_| / log_10_ BCF_observed_, calculated for each BCF value. PSOs were executed 1000 times for each chemical and terminated upon finding a relative error less than 10^− 3^, providing *F*_*gill*_ and *k*_*m*_ that gave highly accurate predictions of a single chemical, but not for any others (Fig. 3c, blue points). Optimized parameter values exhibit a sigmoid-like correlation similar to values optimized across all chemical data (Fig. 3a vs. b). The median of chemical-dependent optimizations (Fig. 3c, black points) can be modeled empirically:

4 $$ {F}_{gill}={F}_{gill}^{\mathrm{max}}\frac{k_m}{K+{k}_m} $$

wherein *F*_*gill*_ = 0.327 μL/s and *K* = 0.00161 s^− 1^ (R^2^ = 0.993). Therefore, a single “effective” metabolic decomposition rate can be determined through experimentation by measuring *F*_*gill*_ and solving Eq. (4) for *k*_*m*_.

Equation (4) lies very close to the correlation obtained by optimizing *F*_*gill*_ and *k*_*m*_ to the entire BCF dataset (Fig. 3c, black line vs. red dots). Therefore, if we parameterize source (*F*_*gill*_) and sink (*k*_*m*_) terms with values taken from Eq. (4) (Fig. 3c, black points), then the resulting model should be reasonably predictive of BCF data across a large variety of nonmetal toxicants. Replacing the individual decomposition rates with Eq. (4) results in a vastly simplified model wherein its simplicity is traded for a reduced predictive accuracy for any individual chemical. One advantage of Eq. (4) is that it completely eliminates the need to parameterize the intracellular metabolic decomposition rate of any organic molecule through additional experimentation, such as hepatocyte assays or studies with primary liver tissues [31], which opens the door to a purely in silico model for predicting BCF in adult zebrafish.

Due to the nature of experimentation, some level of uncertainty can be inherently attributed to all parameter value measurements. We evaluated the robustness of BCF predictions to such uncertainty by randomly sampling each parameter value from a log_2_ normal distribution centered about its literature-derived value and a variance chosen so that samples most often fell within half to twice the literature value (unit variance). We then determined the RMSE for the model by setting *F*_*gill*_ and *k*_*m*_ to the highest scoring values, then sampling a value for the body parameter being tested at random from the lognormal distribution just described. This sampling procedure was repeated 1000 times. If the RMSE value for the optimized model using all literature values for all non-source/sink body parameters was outside the range of 95% of the RMSE values obtained by randomizing the body parameter, then we would conclude that the optimized model was sensitive to variations in that parameter. None of the body parameters, however, had a significant impact on the accuracy of the optimized model.

These results raise an interesting point about the interpretation of *F*_*gill*_ based on its role in the mathematical model. It is usually described as the countercurrent flow rate of water between gill lamellae. Its literature value is 9.167 μL/s [11]. However, choosing a single value for metabolic decomposition restricts its value to lie approximately between 10^− 6^ to 10^− 3^ μL/s (Fig. 3a, white dots); even so, its largest value is approximately 1 uL/s (Fig. 3a and c), far below its literature value. Are these smaller values biologically reasonable? The rate of the chemical transfer from the environment into arterial blood is determined not solely by *F*_*gill*_, but instead by the flux: (*αF*_*gill*_*/V*_*art*_)*C*_*exp*_, wherein *α* gives the chemical assimilation efficiency calculated from an octanol-water partition ratio; *V*_*art*_ is the volume of the arterial blood; and *C*_*exp*_ is the aqueous exposure concentration. We term the rate at which chemical is brought into the body via gills, *αF*_*gill*_*/V*_*art*_, the influx rate. Its value, calculated for each of the 1000 optimization results for all 76 chemicals in our dataset, fell between 10^− 7^ to 10^− 4^ s^− 1^ (Additional file 1: Figure S8). We propose that a reinterpretation of *αF*_*gill*_*/V*_*art*_ as a membrane permeability of the primary and secondary lamellae attached to the gill filaments is consistent with these findings. For example, the overall transport across caco-2 cell membranes of the steroid testosterone (log_10_*K*_*ow*_ = 3.3) can be calculated as approximately 3.44 × 10^− 6^ s^− 1^ by assuming an exchange surface area for zebrafish larvae gills (Additional file 1: Figure S8, blue line) [32, 33], which falls within this range. Although smaller values may be consistent with data, the effect of a larger decomposition rate from, e.g., more decomposition pathways, may elicit a larger water flow and chemical uptake into the fish, and cannot be excluded. Overall, this single decomposition rate approach allows for novel insights into critical physiological processes that drive bioaccumulation, yielding plausible BCF predictions while retaining the biologically relevant parameter estimates.

### Extending reverse toxicokinetic modeling to life stages and species beyond adult zebrafish

**Fig. 4:**
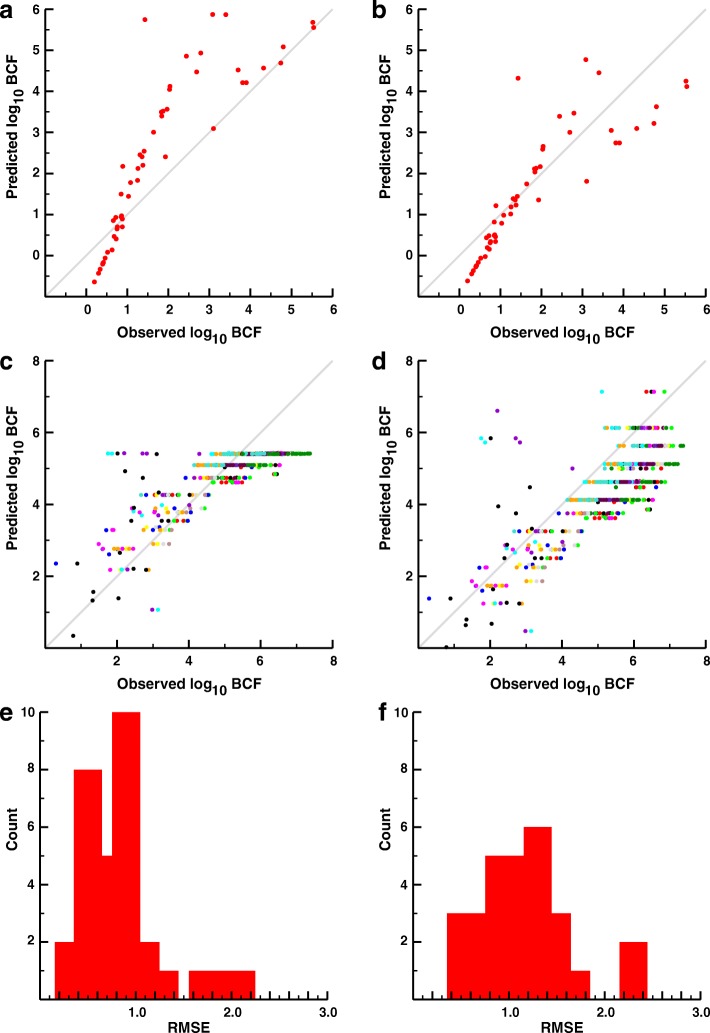
Applicability of the rTK models trained in adult zebrafish to other life-stages of zebrafish and to other fish species. **a** The predicted log_10_ BCF vs. the observed log_10_ BCF values of 55 chemicals measured in zebrafish embryos using the optimized source and sink 7C model (RMSE = 1.220). **b** The predicted log_10_ BCF vs. the observed log_10_ BCF values measured in zebrafish embryos using the 1C model (RMSE = 0.812). **c** The predicted log_10_ BCF values vs. the observed log_10_ BCF values of 97 chemicals measured in 31 species of fish (653 total measurements) using the optimized source and sink 7C model. **d** The predicted log_10_ BCF values vs. the observed log_10_ BCF values from different species using the 1C model. **e** Histogram depicting the number of species with RMSE values for the predicted vs. observed BCFs within a range of values (bin size = 0.2) for the optimized source and sink 7C model. **f** Histogram depicting the number of species with RMSE values for the predicted vs. observed BCFs within a range of values (bin size = 0.2) for the 1C model. The 7C model demonstrates an improvement in the predictability of the BCF values over the 1C model (*p* = 0.0078, Student’s t-test), however, both models demonstrate 95% confidence intervals for RMSE above zero (0.687–1.0 for 7C, 0.99–1.32 for 1C)

We trained and validated the 7C rTK model using log_10_ BCF for 76 chemicals measured in adult zebrafish, but to what extent can it be used to predict BCFs in different life-stages of zebrafish (i.e., embryo) or in other teleosts? To address this question we collected two additional BCF datasets. The first reports log_10_ BCF data for 55 chemicals measured in zebrafish embryos (Additional file 1: Figure S9 and Additional file 2: Table S2) [34–41]. We used the 7C model to predict BCF data by calculating the ratio of body burden to aqueous exposure, which is moderately predictive of embryos (Fig. 4a, RMSE = 1.220) and probably due to the wide range of more hydrophobic chemicals in the dataset (Additional file 1: Figure S9).

This failing of the 7C model should not be surprising, because the morphogenesis of primary organs is not complete until approximately 48 h after fertilization during the hatching period [42]—a multi-tissue compartmentalized modeling approach erroneously reflects the internalized structure of the zebrafish embryo. In contrast, the simpler 1C model is more physiologically relevant, and performed better (RMSE = 0.812) than the optimized 7C model (Fig. 4b).

Can the adult zebrafish models be used to predict BCF data from other physiologically similar fish species? We addressed this question by constructing a dataset for log_10_ BCF values from a variety of chemicals measured in 31 different species of fish, totaling 653 unique data points (Additional file 1: Figure S10 and Additional file 2: Table S3) [28]. These data were compared against predictions from the optimized 7C and the 1C models. On the whole, the 7C model performed better than the 1C across all 31 species (Fig. 4c (7C) and d (1C), RMSE = 0.863 and 1.230). We calculated RMSE values specific to each species (Fig. 4e and f). Although predictions for many species were relatively inaccurate, the 7C model outperformed the 1C model with an average species-specific RMSE of 0.843 versus the 1C’s species-specific RMSE of 1.154. It is likely, assuming similar physiologies between species, that the values of the body parameters, which likely greatly differ from the zebrafish values more than the tested distributions, could be further optimized to provide more accurate results from each of the models.

## Discussion

Often the goal of reverse toxicokinetic modeling is to provide a mathematical framework from which to elicit chemical exposure thresholds correlated with an adverse effect seen in the tissues or body of an organism. Both physiologically based (PBTK) and reverse toxicokinetic (rTK) modeling have been used pragmatically in IVIVE efforts to proactively comply with REACH standards [43] that promise “…to reduce costs and to reduce testing on vertebrate animals” with models parameterized using less invasive and expensive in vitro data. These efforts encompass a more fundamental problem of correlating molecular interactions within the cells of organs and tissues with adverse effects at the whole-organism level, wherein lies a great challenge of accounting for the many rate-limiting chemical transport and decomposition processes regulating tissue bioconcentration.

As a guiding principle, such models ought to minimize the number of parameters obtained at the expense of living systems. To this end we developed rTK models of varying sophistication and physiological fidelity in which chemical-specific biological processes could be replaced using universal constants to eliminate a need for parameterizing experiments to predict the effects of novel compounds on the teleost zebrafish (*Danio rerio*), and to eliminate the need for retraining to new chemical data. Our models take only chemical-specific octanol-water partition ratios as input data, which admits the possibility of leveraging entirely in silico methods, such as quantitative structure-activity relationships or QSARs, to derive bioconcentration factor predictions from knowledge of the chemical structure alone. This aspect alone opens the door to many new possibilities for advancing high-throughput chemical screening efforts.

It was surprising to us that a model parameterized using just a single value for the rate of chemical decomposition, which quantifies chemical-dependent biotransformation, conjugation, and degradation processes within the body [24], could predict bioconcentration factors over such a broad range of chemical compounds. This should be contrasted with the majority of analytic toxicokinetic models that incorporate biochemical processes specific to just a single chemical, or those that use experimentation to establish kinetic rate constants for different chemicals that can all be modeled by identical decomposition kinetics [3–14]. Our models convincingly suggest that training just two parameters to a broad ensemble of chemicals may be a crucial step in facilitating the role of PBTK and rTK modeling in next-generation IVIVE methods that promise a fast, inexpensive first glance at the potential hazards of toxicants or efficacy of new drugs [2–4, 22, 44–48]. These results, however, may be due to the chemicals present in the dataset. Many of the entries are for phenols, polychlorinated biphenols, anilines, benzenes, benzonitriles, and benzenamines with different R-groups (Additional file 2: Table S1). Future studies could test the applicability of the median parameter values to a wider variety of chemical species.

In Eq. (4) we identified a species-specific curve relating a biophysical water-flow parameter in gills (source), which modulates the accessibility of a chemical to the exchange area of the lamellar membranes in gills, to a universal rate of metabolic chemical decomposition (sink), in higher rates erode the availability of chemical within specific tissues of the body. This equation allows for the rate of chemical influx to be finely adjusted according to the rate of efflux from the body. We found that source and sink parameters of the more complex 7C model can be optimized to predict the BCFs of chemicals with octanol water-partition ratios that vary over approximately two orders of magnitude with high statistical agreement with measured data. However, we found that the predictive capability of this model is limited to the age and species of the organisms from which the training data was collected. For example, we showed that our models more poorly predicted BCFs for embryonic zebrafish exposures than for the adult zebrafish data on which it was trained. This trend extends to other teleosts, limiting the ubiquity of the model and suggesting that such rTK models should be developed from species-specific data.

Figure 3 and Eq. (4) relate a chemical independent quantity, *F*_*gill*_, the flow-rate across gill lamellae, to a fundamentally chemical-dependent quantity, *k*_*m*_, the metabolic decomposition rate in body tissues. As we pointed out above, the role of *F*_*gill*_ is to enhance the accessibility of an aqueous chemical to the exchange surface area of gill membranes; the chemical flux reaching arterial blood, *F*_*gill*_*(α/V*_*art*_*)C*_*exp*_ (in the 7C model), increases in proportion to *F*_*gill*_. However, *F*_*gill*_ does not uniquely set this water-to-blood flux, but rather is determined by an assimilation efficiency that varies as a function of the octanol-water partition ratio, *K*_*ow*_. This flux is thus determined according to the identity of dissolved chemical in contact with gill membranes. PSOs indicate that *F*_*gill*_ provides the best statistical agreement with the BCF data across all chemicals with a value at least 4 orders of magnitude smaller than its literature-derived value (Fig. 3a) [11]. If we put this smaller value into the kinetic rate constant *αF*_*gill*_*/V*_*art*_, it falls into a range consistent with the passive transport of larger molecules measured across the lipid bilayer of caco-2 cells [32]. This agreement suggests that smaller values of *F*_*gill*_ are biologically relevant and begs a shift in emphasis from the *F*_*gill*_ term itself to more detailed modeling of the factors which contribute to gill membrane-limited transport of dissolved toxicants into the arterial blood of teleosts. For example, the convoluted structure of gill lamellae probably influence accessibility of chemical to membranes, and since uptake is a boundary-valued process, a fractal geometry [49] might ostensibly contribute to the current value of the lumped assimilation efficiency parameter in our model. This reinterpretation of the source flux is appealing as it describes the distribution of chemical throughout the body not only on a physiological scale, but on a cellular scale as well. Developing standardized multi-scale modeling practices would allow the models to better translate to the challenges posed by IVIVE and encourages the further use of PBTK and rTK models in future systems biology studies.

## Conclusions

Our work suggests a type of universality in the PBTK and rTK models that allows just a single value for the metabolic decomposition rate constant to accurately predict the accumulation of a wide range of chemicals within animal tissues, or equivalently, to predict exposure concentrations from tissue level measurements. While our models may not predict BCFs with the accuracy of other approaches, the ability to model the distribution of chemical between various tissues provides additional capabilities to the modeling platform over other in silico tools to predict BCF, such as QSAR and QSPR [50–54]. In fact, the optimized 7C model, with an RMSE of 0.791, is on par with existing QSAR and QSPR models with reported RMSEs of 0.6 to over 1.0 [53, 54].

A unique ability our model is to correlate an adverse response at the genetic level to an aqueous exposure of an entire organism through, for example, a point-of-departure (POD) analysis of gene-expression data [55] or benchmark dose levels [56] to extrapolate back toward estimation of toxicity threshold levels in environmental exposures. Additionally, our optimized 7C model provides a solid foundation for standardizing a PBTK/rTK modeling pipeline relevant to chemical disposition for different life-stages and species, especially for applications aimed at high-throughput chemical screening or risk and hazard assessment practices. Thus, standardizing PBTK model design, development, and validation procedures across a broad range of terrestrial and aquatic species would not only ensure consistency in future studies, but also help to advance faster and more efficient protocol development relevant to ecotoxicological risk and hazard assessment and human health and drug safety regulations.

To this end, we have explored the limits of generalizing the zebrafish physiology and chemical identity in a way that remains useful; our models maintain zebrafish physiological structure and zebrafish specific parameter values leveraged from measured data. Our results indicate that these models can tolerate a great deal of uncertainty surrounding chemical decomposition rates, yet remain reasonably predictive and potentially useful for chemical screening applications.

## Methods

### Model descriptions

We have based the 7C model on a previously published PBTK model of zebrafish by Pery et al. [11, 19]. The model abstracts the arterial blood, venous blood, brain, gonads, liver, poorly perfused tissues, and richly perfused tissues into 7 distinct components (Fig. 1b). The 1C model combines these tissues, representing the body as a single compartment and is based on previously published models that abstract the human body as a single compartment chemical reactor (Fig. 1a) [21, 22]. In order to simplify the analyses, we previously made two assumptions: 1) exposure concentrations are constant; 2) chemical degradation can be modeled using mass-action kinetics. In prior work we have demonstrated that the mass-action chemical degradation kinetics did not significantly alter the behaviors of the model [19]. These assumptions allow for the derivation of an exact analytical solution for the predicted exposure concentration as a linear function of whole body concentration, making the predictions of BCFs by the model relatively straightforward. Details on the models and their parameters can be found in Section “Discussion” of the Supplementary Information.

### Particle swarm optimizations

We used PSOs to find parameter sets that provided accurate estimations of BCFs for different chemicals [57]. For each model’s PSO we indicate the parameters to be optimized and their bounds. In order to avoid bias we only set the condition that all body parameters must be positive. The PSO then initializes 1000 particles – each particle represents a set of values for the parameters to be optimized – by randomly setting initial values for the parameters. The particles are then scored based on how accurately a model parameterized by their values predicts BCFs for a set of chemicals by calculating the R^2^ for the predicted vs. observed points for the line y = x. Velocities for each particle within the parameter space are computed based on their previous velocities, their position relative to their best scoring position, and their position relative to the best scoring position across all particles. The positions of the particles – the values of the parameters in each set – are then updated based on the new velocities. The particles are then rescored and updated until one reaches a score above the indicated cutoff.

## Additional files

Additional file 1: Supporting Information includes all Supporting Figures and a complete description of the derivation and parameterization of the models used in this work. (PDF 734 kb)

Additional file 2: **Table S1** includes log_10_ K_ow_ values and log_10_ BCF values for various chemicals as measured or derived from measurements taken in adult zebrafish. **Table S2** Table S2 includes log_10_ K_ow_ values and log_10_ BCF values for various chemicals as measured or derived from measurements taken in embryonic zebrafish. **Table S3** Table S3 includes log_10_ K_ow_ values and log_10_ BCF values for various chemicals as measured or derived from measurements taken in various other fish species. (XLSX 81 kb)

## Abbreviations

1C: 1-compartment
7C: 7-compartment
ADME: Absorption, distribution, metabolism, and excretion
BCF: Bioconcentration factor
IVIVE: In vitro to in vivo extrapolation
PBTK: Physiologically based toxicokinetic
PSO: Particle swarm optimization
rTK: Reverse toxicokinetic
Tox21: Toxicology in the twenty-first Century
