# Supplementary material for: Statistical relationship between metabolic decomposition and chemical uptake predicts bioconcentration factor data for diverse chemical exposures
Source: BMC Syst Biol. 2018 Aug 7;12:81. doi: 10.1186/s12918-018-0601-y (PMC6081876; doi:10.1186/s12918-018-0601-y)
Supplement: Supplementary file 1 — Supporting Information includes all Supporting Figures and a complete description of the derivation and parameterization of the models used in this work. (PDF 734 kb) [file 12918_2018_601_MOESM1_ESM.pdf]

Statistical relationship between metabolic decomposition and chemical uptake predicts bioconcentration factor data for diverse chemical exposures

Supplemental Information

Michael A. Rowland[1,2], Hannah Wear[3], Karen H. Watanabe[4], Kurt A. Gust[1], and Michael L. Mayo[1]

[1] Environmental Laboratory, U.S. Army Corps of Engineers, Halls Ferry Road, Vicksburg, MS, USA
[2] Oak Ridge Institute for Science and Education, Oak Ridge, TN, USA
[3] Portland State University, Portland, OR, USA
[4] School of Mathematical and Natural Sciences, Arizona State University, Glendale, AZ, USA

Email: Michael.L.Mayo@usace.army.mil;

Contents

1 Supplemental Figures **2**

2 All Parameter Particle Swarm Optimization **11**

3 Models **13**

3.1 7-Compartment rTK Model 13

3.2 1-Compartment rTK Model 18

1 Supplemental Figures

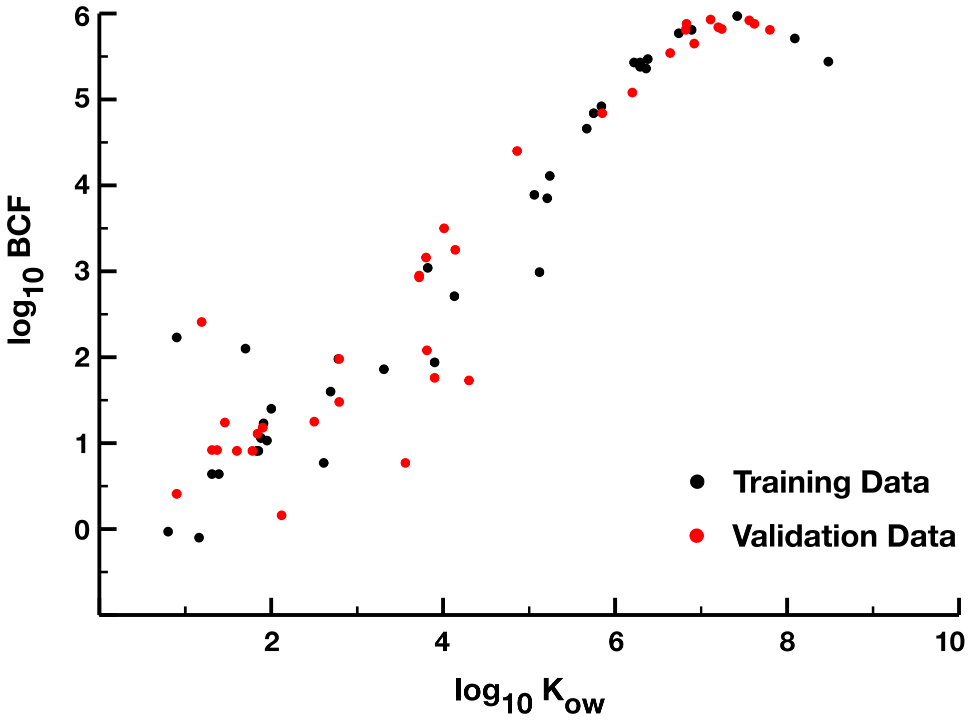

Figure S1: The $\log_{10}$ BCF values vs. their associated $\log_{10}$ K_{ow} values for 76 chemicals measured in adult zebrafish [Fox et al., 1994, Butte et al., 1991, Min and Cha, 2000, Mukhi and Patino, 2008, Arnot and Gobas, 2006, Wang et al., 2015, Cha and Bae, 2014, Zok et al., 1991]. Black dots are data points that have been randomly sorted into the training dataset while red dots are data points that have been randomly sorted into the validation dataset. See Table S1 for exact values and associated references.

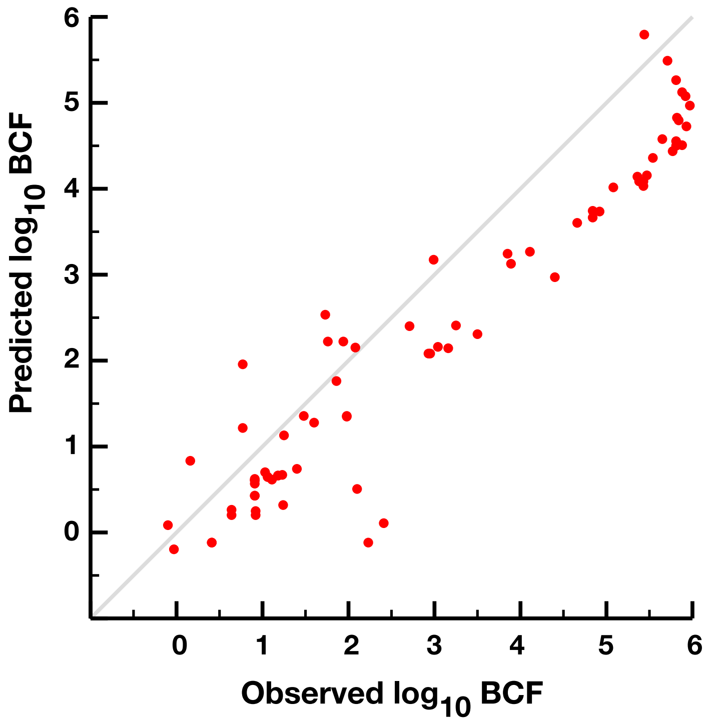

Figure S2: The $\log_{10}$ BCF values predicted by the 1C model with $k_m = 0$ vs. the associated ob-served $\log_{10}$ BCF for all 76 chemicals. The grey line represents the line y=x and is used to calculate the coefficient of determination of the scatterplot ($R^2 = 0.737$).

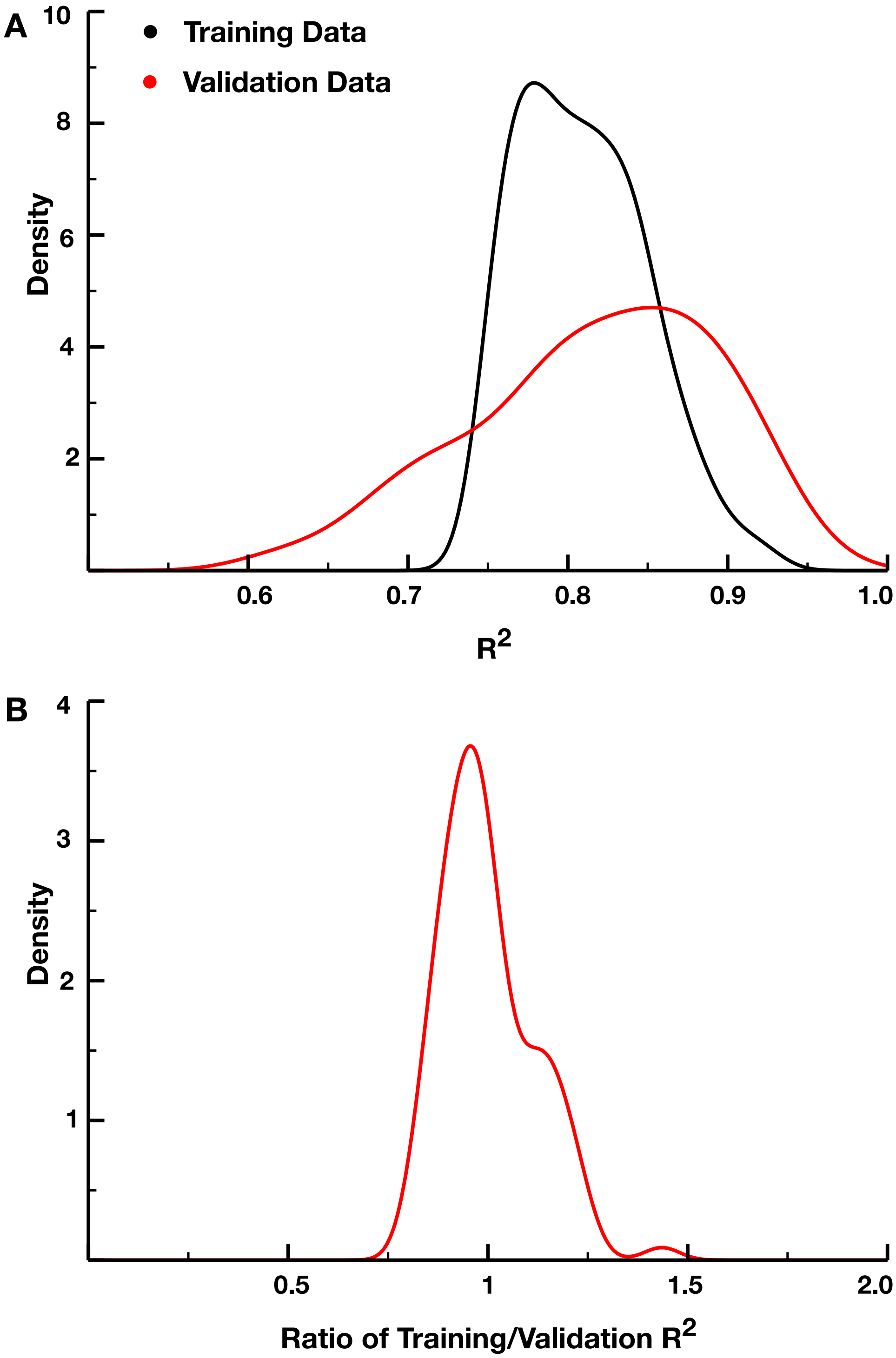

Figure S3: Testing for bias in the random assignments of the training and validation datasets. (A) The distribution of R^2 values obtained from a single training of the 7C model for each of 100 random assignments of the 76 chemicals into training and validation datasets. For each randomization, the 7C model was parameterized based on the results of a single PSO with a cutoff of $R^2 = 0.75$ scored against the training dataset. The plotted R^2 values represent the final scores from the PSOs for the training dataset and the R^2s for the validation set using the 7C models parameterized by the PSOs. There is no evidence rejecting the null hypothesis that the difference in the mean R^2 values is not 0 ($p = 0.2237$, Student's t-test).

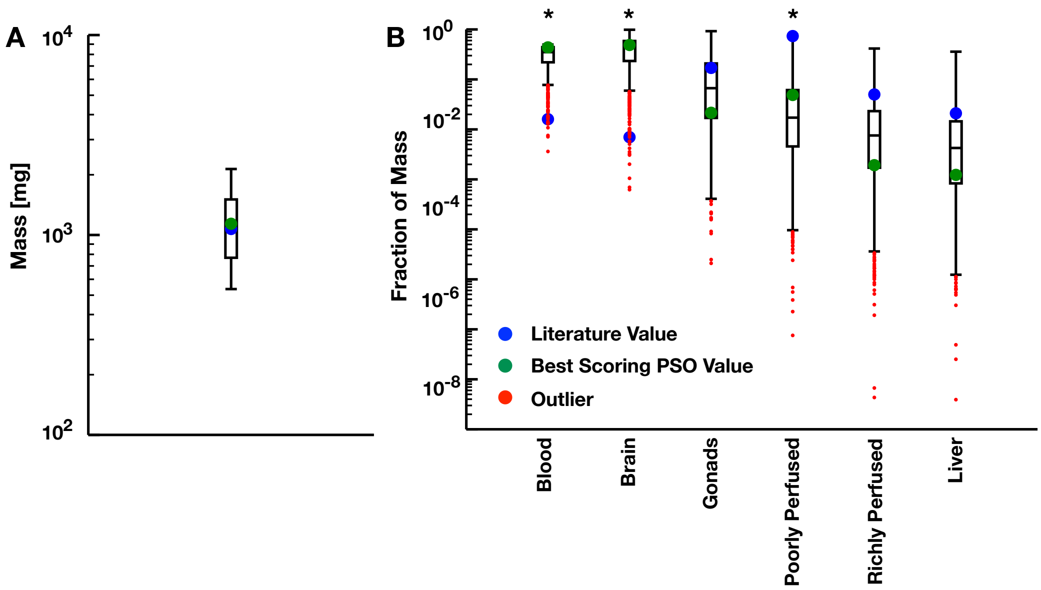

Figure S4: The distribution of values for the total mass (A) and the fraction of the total mass per compartment (B) from the results of 1000 PSOs of the 7C model with cutoff $R^2 = 0.8$. The literature derived values are shown as blue dots and the values from the highest scoring PSO parameter set are shown as green dots. Asterisks highlight parameters in which the literature value lies outside of the range of 95% of the PSO derived values.

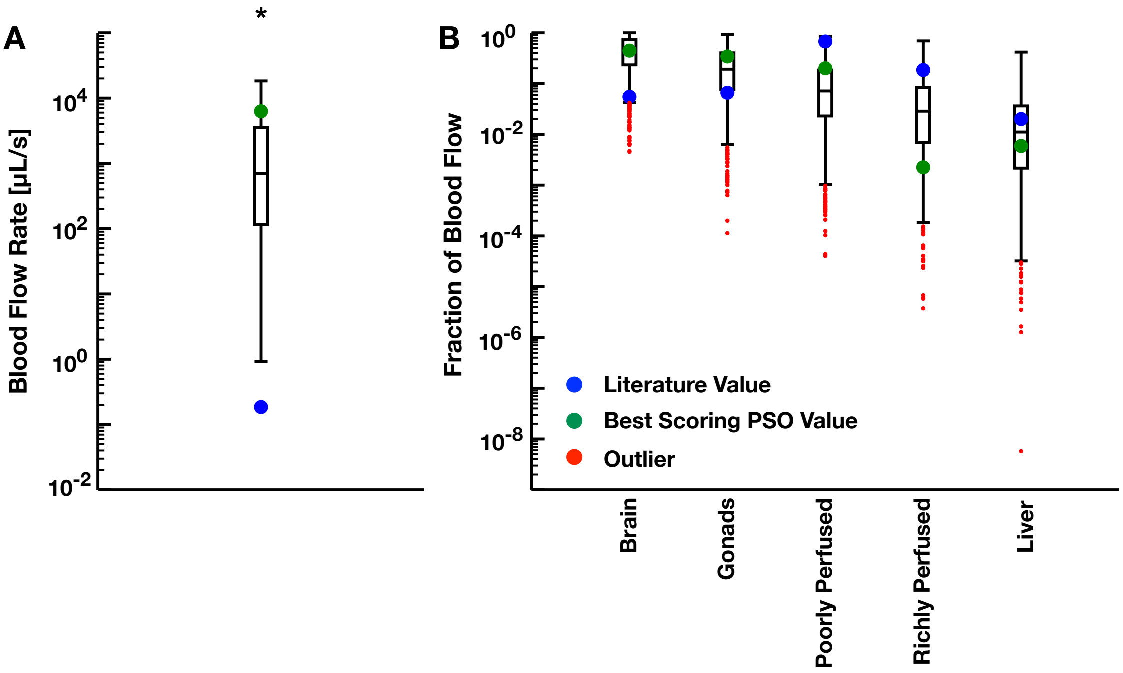

Figure S5: The distribution of values for the total blood flow rate (A) and the fraction of the blood flow per compartment (B) from the results of 1000 PSOs of the 7C model with cutoff $R^2 = 0.8$. The literature derived values are shown as blue dots and the values from the highest scoring PSO parameter set are shown as green dots. Asterisks highlight parameters in which the literature value lies outside of the range of 95% of the PSO derived values.

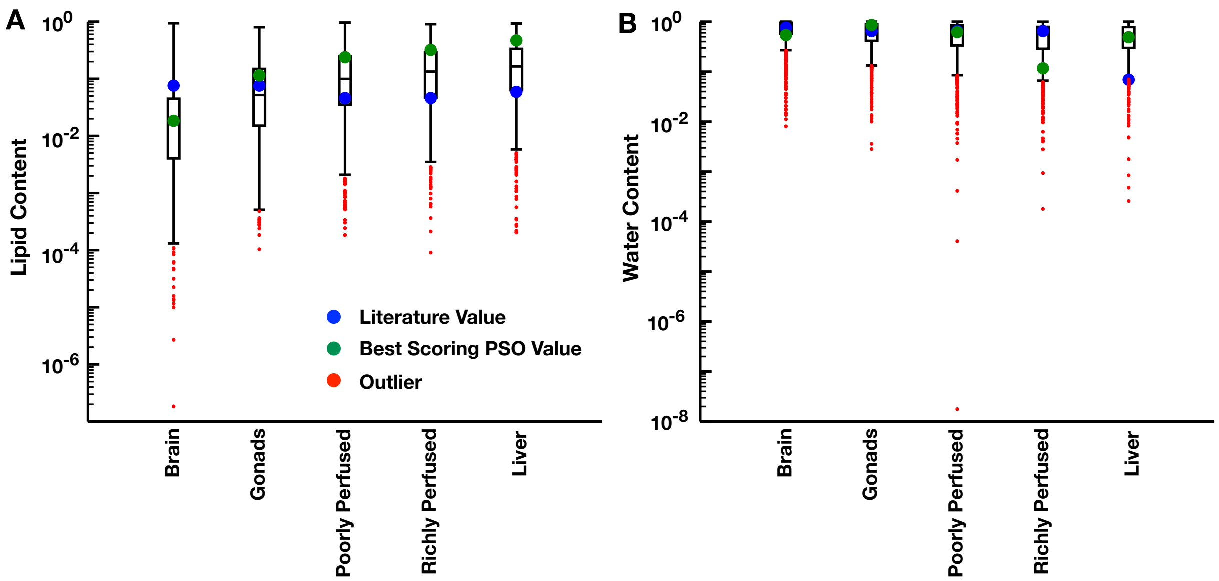

Figure S6: The distribution of values for the lipid content (A) and the water content (B) of per compartment from the results of 1000 PSOs of the 7C model with cutoff $R^2 = 0.8$. The literature derived values are shown as blue dots and the values from the highest scoring PSO parameter set are shown as green dots. Asterisks highlight parameters in which the literature value lies outside of the range of 95% of the PSO derived values.

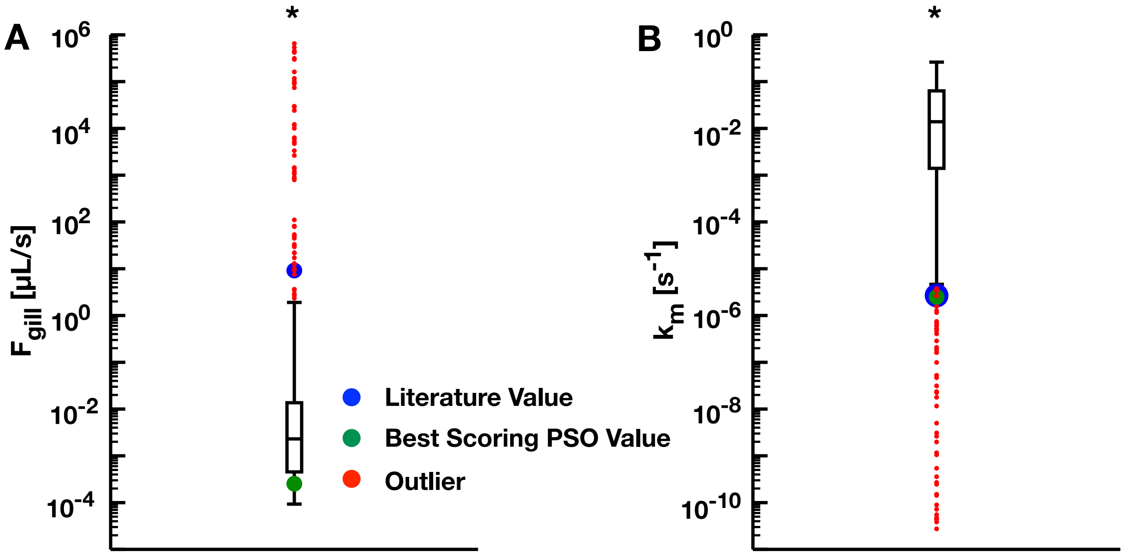

Figure S7: The distribution of values for the water flow rate through the gills (A) and the rate for the metabolism of chemicals (B) from the results of 1000 PSOs of the 7C model with cutoff $R^2 = 0.8$. The literature derived values are shown as blue dots and the values from the highest scoring PSO parameter set are shown as green dots. Asterisks highlight parameters in which the literature value lies outside of the range of 95% of the PSO derived values.

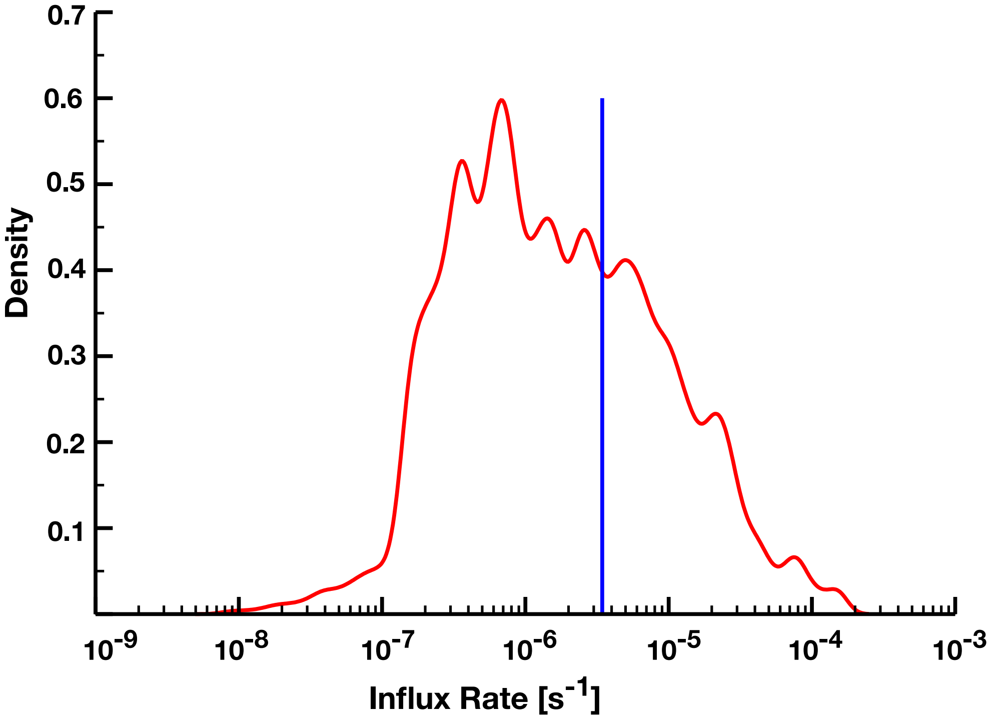

Figure S8: The distribution of influx rates for all 76 chemicals based on the 1000 values for F_{gill} determined by optimizing F_{gill} and k_m for the 7C model. The influx rates were determined by calculating the assimilation efficiency factor (α) for each chemical, which was then multiplied by F_{gill} / V_{art}, where V_{art} is the volume of the arterial blood. The blue line shows the estimated influx rate of testosterone (3.44 x 10^{-6} s^{-1}), based on the influx permeability of caco-2 membranes and the potential exchange surface of zebrafish larvae gills [Hilgers et al., 1990, Rombough, 1999].

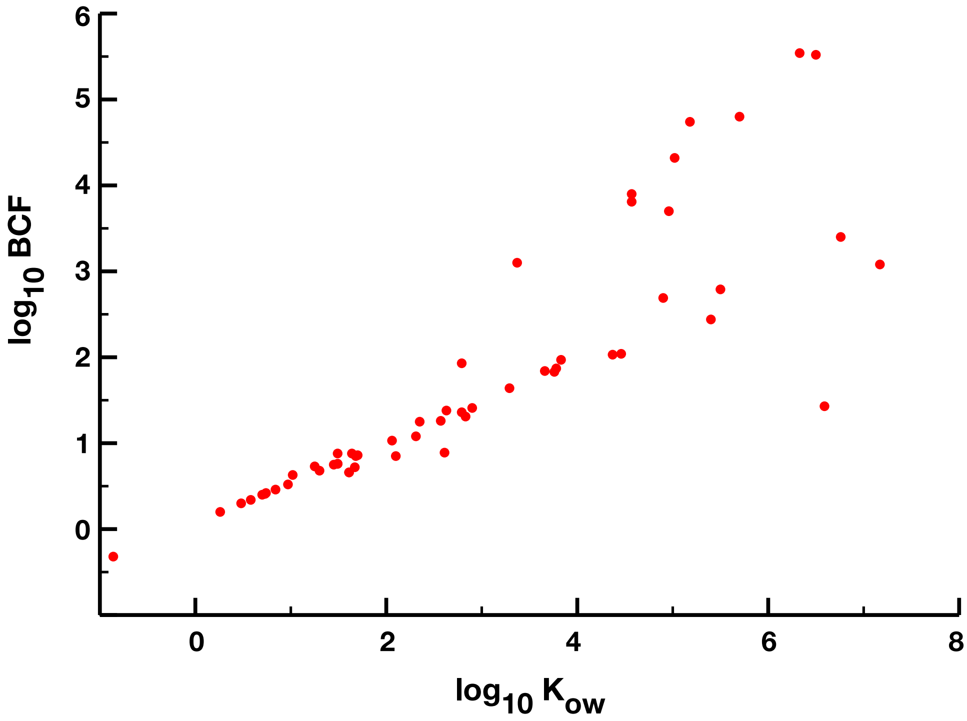

Figure S9: The $\log_{10}$ BCF values vs. their associated $\log_{10}$ K_{ow} values for 55 chemicals measured in zebrafish embryos [El-Amrani et al., 2012, Schreiber et al., 2009, Liu et al., 2015, Stanley et al., 2009, Petersen and Kristensen, 1998, Tu et al., 2014, Hertl and Nagel, 1993, Brust, 2001]. See Table S2 for exact values and references.

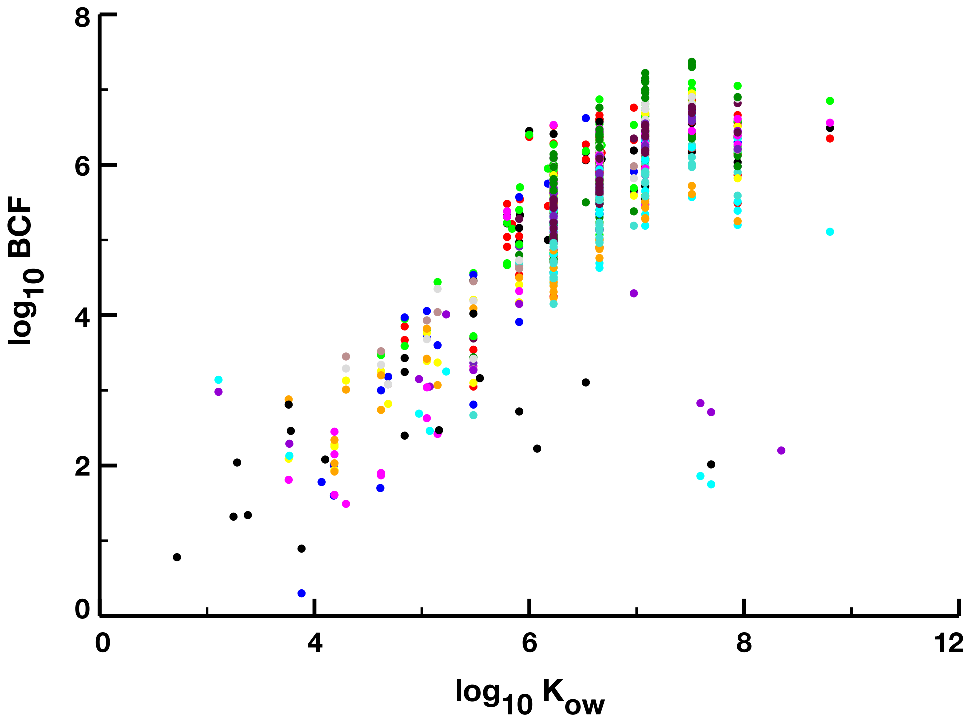

Figure S10: The $\log_{10}$ BCF values vs. their associated $\log_{10}$ K_{ow} values for 97 chemicals measured in 31 fish species (653 total points) [Arnot and Gobas, 2006]. See Table S3 for exact values and references.

2 All Parameter Particle Swarm Optimization

In our first attempts at optimizing the 1C and 7C models, we initially did a blind set of PSOs in which we allowed all physiological parameters to vary. PSOs of the 1C model that were terminated using an $R^2 = 0.8$ cutoff could not provide parameter values that improved the fit. In addition, parameter values from PSOs completed using a fixed number of updating steps also resulted in no discernible increase in accuracy, providing BCF predictions identical to those obtained with the literature-derived parameters values. The 1C model consistently underestimated the measured BCF data (Fig. S11A, RMSE = 0.894 and 0.924 (training and validation), 66/76 underestimated, p=2.96 x 10^{-11}, binomial test).

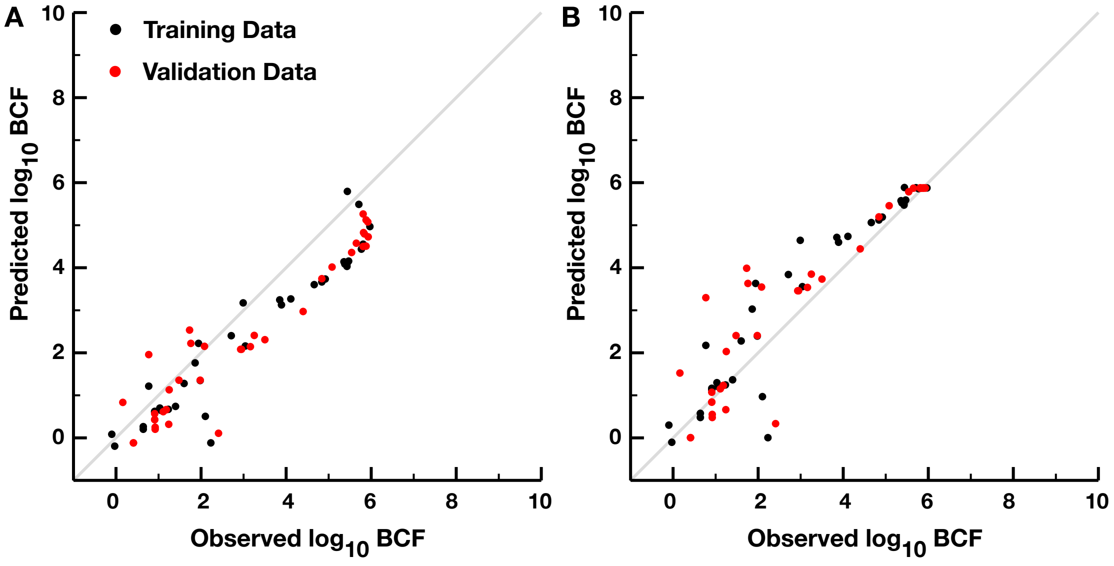

Figure S11: Predicted vs. observed BCFs for optimized 1C and 7C models. (A) The predicted $\log_{10}$ BCF values estimated by the 1C model from the $\log_{10} K_{ow}$ values for each of the chemicals in the training and validation sets vs. the observed $\log_{10}$ BCF values fter optimizing the model with a specified number of PSO updates. (B) The predicted $\log_{10}$ BCF valeus estimated by the 7C model from the $\log_{10} K_{ow}$ values for each of the chemicals in the training and validation sets vs. the observed $\log_{10}$ BCF values after optimization.

We then executed the PSO 1000 times for the 7C model, wherein particle update steps were terminated upon reaching an optimization target of at least $R^2 = 0.8$ for fitting the predicted vs. optimized BCFs to the line y=x. Figure S11B illustrates the 7C model using the highest scoring parameter set out of the 1000 optimizations described above. With these optimized parameters, the 7C model was able to predict the BCFs for the training and validation data sets with a higher degree of accuracy (RMSE = 0.728 and 0.850). The optimized 7C model, though, still significantly overestimates the BCF data (19/76 overestimated, p=1.48x10^{-5}, binomial test).

A model trained using a biased dataset will be less predict than one trained on a dataset that captures the intrinsic variation of the full dataset, a problem referred to commonly as sampling

bias and is a form of selection bias. To determine the extent of such bias in the data subsets used to train and test our models, we randomly selected new training and validation sets from the whole of the BCF dataset 100 times, each time retraining the 7C model to the new training dataset using a PSO cutoff of $R^2 = 0.75$. The R^2 values were recalculated for both datasets (Fig. S3A). None of the 100 training or validation datasets exhibited R^2 values that were significantly different from our earlier choice (Fig. S3B, 41/100 validation R^2s being greater than the training R^2, p = 0.09, binomial test). In addition, there was also no correlation between training and R^2 and validation R^2 (Pearson's r = -0.071). Based on these results we conclude that differences between the R^2 values calculated from the 7C model optimized using our original training and validation datasets did not result from any inherent bias in the selection procedure, and that these results are robust across different partitions of the dataset.

We imposed to restrictions on parameter values identified from PSOs carried out using the 100 different training dataset partitions; there is, therefore, no guarantee that optimal parameter values be physically plausible. Parameters modified by the PSOs include the total body mass and the distribution of mass between compartments (Fig. S4), total blood flow rate and the distribution of the blood flow between compartments (Fig. S5), the lipid and water contents of each compartment (Fig. S6), and the water flow rate through the gills and metabolic rate of the chemicals (Fig. S7). In each figure we not only show the distribution of values of the parameters across the optimizations, but the literature-derived values (blue) and the values from the highest scoring optimization (green). Additionally, we marked parameters in which the literature values are outside of the range of 95% of the optimization values with an asterisk.

We find that optimized blood and brain masses are significantly larger than literature values, with brain tissues often representing at least 40% of the body's mass (Fig. S4B). Optimized masses of poorly and richly perfused tissues exhibit substantially smaller values than reported in literature. Additionally, the optimized blood flow rate to poorly perfused tissues is smaller than its pre-optimization value (Fig. S5B). Finally, optimized lipid content of brain tissues is smaller by at least an order of magnitude when compared to literature data (Fig. S6A). It is likely that the focus of the PSOs on these compartments may come from their initial values, presenting the PSOs a path of least resistance towards reducing the overall lipid content of the fish body in order to reduce the accumulation of lipophilic chemicals.

Some model-optimization results are nonsensical and highlight the benefit of literature data for comparison. For example, optimized parameter values indicate that fish host a brain 40% of body mass with less than 2% of the brain mass being lipids, and that blood contributes much of the remaining body mass. Obviously such parameter values are anatomically/physiologically unrealistic. The optimized parameters, with a relatively low total lipid content, describe a system that would be poorly suited for retaining lipophilic chemicals. Thus, whether a model is suitable depends not only on its predictive performance, but also on its biological relevance. These discrepancies point to a general tendency for PSOs to produce smaller total body concentrations of the chemicals, thus achieving their goal of reducing the predicted BCFs. The pre-optimized 7C model overestimated the BCFs of the chemicals; an overestimate of the total body concentration of the chemical by the body would result in higher BCF values. The PSO optimized model parameters created a system that would hold onto less chemical, yielding more reasonable predictions of BCFs. The PSOs, however, generated non-biologically relevant parameter estimates

for well-established values (i.e. brain to body proportions and brain lipid content).

3 Models

3.1 7-Compartment rTK Model

We have previously derived an exact analytical solution describing the predicted exposure concentration as a function of the total body concentration of a chemical when the body concentration is at steady state with the environment:

$$C_{H_2O} = C_{tot}/V_{tot}/\left(\sum_i V_i/A_i\right) \tag{S1}$$

Where $i = brn, gon, ppt, rpt, liv, art, ven$. C_{tot} is defined as the total body concentration of the chemical, V_{tot} is the total volume of the organism, and C_{H_2O} is the predicted exposure concentration [Rowland et al., 2017]. We defined A_i as:

$$\begin{aligned}
A_{brn} &\equiv \frac{D_1}{D_2 P_{brn}} \\
A_{gon} &\equiv \frac{D_1}{D_2 P_{gon}} \\
A_{ppt} &\equiv \frac{D_1}{D_2 P_{ppt}} \\
A_{rpt} &\equiv \frac{D_1}{D_2 P_{rpt}} \\
A_{liv} &\equiv \frac{D_1}{D_3 P_{liv}} \\
A_{art} &\equiv \frac{D_1}{D_2} \\
A_{ven} &\equiv \frac{D_1}{D_4}
\end{aligned}$$

Where D_j is:

$$\begin{aligned}
D_1 &\equiv F_{car}(\alpha F_{gill}(F_{gon} + F_{rpt} + F_{liv} + V_{liv} k_m) + P_{bw} V_{liv} k_m (F_{gon} + F_{rpt} + F_{liv})) \\
D_2 &\equiv \alpha F_{gill}(F_{gon} + F_{rpt} + F_{liv} + V_{liv} k_m)(\alpha F_{gill} + P_{bw} F_{car}) \\
D_3 &\equiv \alpha F_{gill}(F_{gon} + F_{rpt} + F_{liv})(\alpha F_{gill} + P_{bw} F_{car}) \\
D_4 &\equiv \alpha F_{gill}((F_{gon} + F_{rpt} + F_{liv}) F_{car} + V_{liv} k_m (F_{brn} + F_{ppt})) P_{bw}
\end{aligned}$$

F_{car} is the total blood flow rate, F_{gon}, F_{rpt}, and F_{liv} are the blood flow rates through each of the indicated compartments, P_i is the tissue:blood partition ratio for the indicated compartments, P_{bw} is the blood:water partition ratio, V_{liv} is the volume of the liver, and α is the assimilation efficiency of the chemical.

The body-specific parameters include:

Parameter	Description	Value	Unit
F_{brn}	Blood flow rate through brain	0.010175	μL/s
F_{gon}	Blood flow rate through gonads	0.01221	μL/s
F_{ppt}	Blood flow rate through poorly perfused tissues	0.12469	μL/s
F_{rpt}	Blood flow rate through richly perfused tissues	0.034225	μL/s
F_{liv}	Blood flow rate through liver	0.0037	μL/s
V_{art}	Volume of arterial blood	5.716	μL
V_{ven}	Volume of venous blood	11.431	μL
V_{brn}	Volume of brain	7.502	μL
V_{gon}	Volume of gonads	182.189	μL
V_{ppt}	Volume of poorly perfused tissues	788.771	μL
V_{rpt}	Volume of richly perfused tissues	53.585	μL
V_{liv}	Volume of liver	22.506	μL
F_{gill}	Volumetric flow rate of water through the gills	9.167	μL/s

The values for the blood flow in the different organs are calculated as:

$$\begin{aligned}
F_{brn} &\equiv f_{brn} f_{tot} \\
F_{gon} &\equiv f_{gon} f_{tot} \\
F_{ppt} &\equiv f_{tot} - F_{brn} - F_{gon} - F_{rpt} - F_{liv} \\
F_{rpt} &\equiv f_{rpt} f_{tot} \\
F_{liv} &\equiv f_{liv} f_{tot}
\end{aligned}$$

Where f_{tot} is the total blood flow rate throughout the body and f_i are the scaled blood flow rates for each tissue compartment. The values for the volume of the compartments are calculated as:

$$\begin{aligned}
V_{art} &\equiv 0.33 \frac{m_b m_{tot}}{\rho} \\
V_{ven} &\equiv 0.67 \frac{m_b m_{tot}}{\rho} \\
V_{brn} &\equiv \frac{m_{brn} m_{tot}}{\rho} \\
V_{gon} &\equiv \frac{m_{gon} m_{tot}}{\rho} \\
V_{ppt} &\equiv \frac{(1 - m_b - m_{brn} - mgon - m_{liv} - m_{rpt}) m_{tot}}{\rho} \\
V_{rpt} &\equiv \frac{m_{rpt} m_{tot}}{\rho} \\
V_{liv} &\equiv \frac{m_{liv} m_{tot}}{\rho}
\end{aligned}$$

where the value of the average density of the fish's body, ρ, is assumed to be 1 mg/μL. Finally,

the value for F_{gill} is a simple scaling of f_{wat} to appropriate units:

$$F_{gill} = f_{wat} \times 10^3$$

Parameters given by Péry, *et al.* [Pery et al., 2014] include:

Parameter	Description	Value - male	Value - female	Unit
f_{car}	Cardiac output - (0.5g/26°C)	11.1	11.1	μL/min
f_{brn}	Scaled blood flow - brain	0.055	-	-
f_{gon}	Scaled blood flow - gonads	0.066	-	-
f_{rpt}	Scaled blood flow - richly perfused tissues	0.185	-	-
f_{liv}	Scaled blood flow - liver	0.02	-	-
m_{tot}	Total mass	617.6	1071.7	mg
m_{bld}	Percentage of total mass - blood	1.6	1.6	-
m_{brn}	Percentage of total mass - brain	1.5	0.7	-
m_{gon}	Percentage of total mass - gonads	1.8	8 or **17**	-
m_{rpt}	Percentage of total mass - richly perfused tissues	5	5	-
m_{liv}	Percentage of total mass - liver	0.8	2.1	-
F_{gill}	Ventilation rate - (0.4g/27°C)	0.55	0.55	mL/min
W_{liv}	Water content - liver	0.65	0.69	-
W_{brn}	Water content - brain	0.75	0.76	-
W_{gon}	Water content - gonads	0.52	0.65	-
W_{tot}	Water content - total	0.68	0.65	-
L_{liv}	Lipid content - liver	0.105	0.059	-
L_{brn}	Lipid content - brain	0.073	0.076	-
L_{gon}	Lipid content - gonads	0.22	0.076	-
L_{oth}	Lipid content - rest of the body	0.046	0.046	-

We assume that the scaled blood flow in males and females are the same. The chemical-specific and simulation parameters include:

Parameter	Description	Units
C_{art}	Chemical concentration in arterial blood	μmol/μL
C_{ven}	Chemical concentration in venous blood	μmol/μL
C_{brn}	Chemical concentration in brain	μmol/μL
C_{gon}	Chemical concentration in gonads	μmol/μL
C_{ppt}	Chemical concentration in poorly perfused tissues	μmol/μL
C_{rpt}	Chemical concentration in richly perfused tissues	μmol/μL
C_{lib}	Chemical concentration in liver	μmol/μL
C_{H_2O}	Chemical concentration in water	μmol/μL
P_{brn}	Partition coefficient between brain and blood	-
P_{gon}	Partition coefficient between gonads and blood	-
P_{ppt}	Partition coefficient between poorly perfused tissues and blood	-
P_{rpt}	Partition coefficient between richly perfused tissues and blood	-
P_{liv}	Partition coefficient between liver and blood	-
k_m	Liver metabolism rate	s^{-1}
α	Assimilation efficiency	-
P_{bw}	Blood:water partition ratio	-

Additional values given by equations:

Description	Value
P_{bw}	$\log(P_{bw}) = 0.78\log(K_{ow}) - 0.82$
P_i	$\log(P_i$ - $W_i) = 0.74 \times \log(K_{ow}) + 1.00 \times \log(L_i) + 0.72$

The 7C model was used in Fig. 2B of the main text. For this figure we used the following physiological parameters:

Parameter	Value
f_{car}	0.185 μL/s
f_{brn}	5.5%
f_{gon}	6.6%
f_{rpt}	18.5%
f_{ppt}	67.4%
f_{liv}	2.0%
m_{tot}	1071.7 mg
m_{bld}	1.6%
m_{brn}	0.7%
m_{gon}	17.0%
m_{rpt}	5.0%
m_{ppt}	73.6%
m_{liv}	2.1%
F_{gill}	9.167 μL/s
L_{brn}	7.6%
L_{gon}	7.6%
L_{rpt}	4.6%
L_{ppt}	4.6%
L_{liv}	5.9%
W_{brn}	76.0%
W_{gon}	65.0%
W_{rpt}	65.0%
W_{ppt}	65.0%
W_{liv}	6.9%

The assimilation efficiency and partition coefficients for chemicals were calculated based on their $\log_{10} K_{ow}$s and physiological parameters according to the previously described relationships. The metabolic decomposition rate was set at 2.679 x $10^{-6} s^{-1}$ for all chemicals. The 7C model was also used in Figures 3 and 4A & C of the main text. In Figure 3A and C, the F_{gill} and metabolic decomposition rates (k_m) were varied as indicated in the figures. in Fig. 3B and Fig. 4A & C we used the values 5.78242 x $10^{-6}\mu$L/s and 2.2271 x $10^{-8} s^{-1}$ for F_{gill} and k_m.

3.2 1-Compartment rTK Model

The 1-Compartment model treats the fish as a single compartment with gills. We have previously derived an exact analytical solution for the predicted exposure concentration as a function of the total body concentration:

$$C_{H_2O} = \frac{(P_{bw}V_{tot}k_m + F_{gill}P_t)C_{tot}}{F_{gill}P_{bw}P_t} \tag{S2}$$

where P_t is defined as a the tissue:blood ratio for the single compartment [Rowland et al., 2017]. The literature-derived parameter values were adapted from those given by Péry, *et al.* [Pery et al., 2014]:

Parameter	Value	Unit
F_{gill}	14.8789577	μL/s
m_{tot}	1071.7	mg
LC_t	0.050847	-
WC_t	0.628169	-
k_m	2.679 x 10^{-6}	s^{-1}

P_t is calculated in the same manner as P_i in the 7C model and P_{bw} is calculated in the same manner as in the 7C model. These parameters were used to generate Figs. 1A and 4B & D of the main text.

References

Arnot and Gobas, 2006. Arnot, J. A. and Gobas, F. A. P. C. (2006). A review of bioconcentration factor (BCF) and bioaccumulation factor (BAF) assessments for organic chemicals in aquatic organisms. Environmental Reviews *14*, 257–297.

Brust, 2001. Brust, K. (2001). Toxicity of aliphatic amines on the embryos of zebrafish Danio rerio - experimental studies and QSAR. Thesis Technische Universitat Dresden.

Butte et al., 1991. Butte, W., Fox, K. and Zauke, G. P. (1991). Kinetics of Bioaccumulation and Clearance of Isomeric Hexachlorocyclohexanes. Science of the Total Environment *109*, 377–382.

Cha and Bae, 2014. Cha, C. and Bae, H. (2014). Effects and Bioconcentration of Dichlorvos and Phosalone on Zebrafish (Brachydanio rerio). Research Journal of Environmental Toxicology *8*, 110–116.

El-Amrani et al., 2012. El-Amrani, S., Pena-Abaurrea, M., Sanz-Landaluze, J., Ramos, L., Guinea, J. and Camara, C. (2012). Bioconcentration of pesticides in zebrafish eleutheroembryos (Danio rerio). Sci Total Environ *425*, 184–90.

Fox et al., 1994. Fox, K., Zauke, G. P. and Butte, W. (1994). Kinetics of Bioconcentration and Clearance of 28 Polychlorinated Biphenyl Congeners in Zebrafish (Brachydanio-Rerio). Ecotoxicology and Environmental Safety *28*, 99–109.

Hertl and Nagel, 1993. Hertl, J. and Nagel, R. (1993). Bioconcentration and Metabolism of 3,4-Dichloroaniline in Different Life Stages of Guppy and Zebrafish. Chemosphere *27*, 2225–2234.

Hilgers et al., 1990. Hilgers, A. R., Conradi, R. A. and Burton, P. S. (1990). Caco-2 cell monolayers as a model for drug transport across the intestinal mucosa. Pharm Res *7*, 902–10.

Liu et al., 2015. Liu, H., Tang, S., Zheng, X., Zhu, Y., Ma, Z., Liu, C., Hecker, M., Saunders, D., Giesy, J., Zhang, X. and Yu, H. (2015). Bioaccumulation, Biotransformation, and Toxicity of BDE-47, 6-OH-BDE-47, and 6-MeO-BDE-47 in Early Life-Stages of Zebrafish (Danio rerio). Environmental Science & Technology *49*, 1823–1833.

Min and Cha, 2000. Min, K. J. and Cha, C. G. (2000). Determination of the bioconcentration of phosphamidon and profenofos in zebrafish (Brachydanio rerio). Bulletin of Environmental Contamination and Toxicology *65*, 611–617.

Mukhi and Patino, 2008. Mukhi, S. and Patino, R. (2008). Effects of hexahydro-1,3,5-trinitro-1,3,5-triazine (RDX) in zebrafish: General and reproductive toxicity. Chemosphere *72*, 726–732.

Pery et al., 2014. Pery, A. R., Devillers, J., Brochot, C., Mombelli, E., Palluel, O., Piccini, B., Brion, F. and Beaudouin, R. (2014). A physiologically based toxicokinetic model for the zebrafish Danio rerio. Environ Sci Technol *48*, 781–90.

Petersen and Kristensen, 1998. Petersen, G. I. and Kristensen, P. (1998). Bioaccumulation of Lipophilic Substances in Fish Early Life Stages. Environmental Toxicology and Chemistry *17*, 1385–1395.

Rombough, 1999. Rombough, P. (1999). The gill of fish larvae. Is it primarily a respiratory or an ionoregulatory structure? Journal of Fish Biology *55*, 186–204.

Rowland et al., 2017. Rowland, M., Perkins, E. J. and Mayo, M. (2017). Physiological fidelity or model parsimony? The relative performance of reverse-toxicokinetic modeling approaches. BMC Syst Biol *11*.

Schreiber et al., 2009. Schreiber, R., Altenburger, R., Paschke, A., Schuurmann, G. and Kuster, E. (2009). A novel in vitro system for the determination of bioconcentration factors and the internal dose in zebrafish (Danio rerio) eggs. Chemosphere *77*, 928–33.

Stanley et al., 2009. Stanley, K. A., Curtis, L. R., Simonich, S. L. and Tanguay, R. L. (2009). Endosulfan I and endosulfan sulfate disrupts zebrafish embryonic development. Aquat Toxicol *95*, 355–61.

Tu et al., 2014. Tu, W., Lu, B., Niu, L., Xu, C., Lin, C. and Liu, W. (2014). Dynamics of uptake and elimination of pyrethroid insecticides in zebrafish (Danio rerio) eleutheroembryos. Ecotoxicol Environ Saf *107*, 186–91.

Wang et al., 2015. Wang, Y., Xu, L., Li, D. Z., Teng, M. M., Zhang, R. K., Zhou, Z. Q. and Zhu, W. T. (2015). Enantioselective bioaccumulation of hexaconazole and its toxic effects in adult zebrafish (Danio rerio). Chemosphere *138*, 798–805.

Zok et al., 1991. Zok, S., Gorge, G., Kalsch, W. and Nagel, R. (1991). Bioconcentration, metabolism and toxicity of substituted anilines in the zebrafish (Brachydanio rerio). Sci Total Environ *109-110*, 411–21.
